# Exposure Therapy With Personalized Real-Time Arousal Detection and Feedback to Alleviate Social Anxiety Symptoms in an Analogue Adult Sample: Pilot Proof-of-Concept Randomized Controlled Trial

**DOI:** 10.2196/13869

**Published:** 2019-06-14

**Authors:** Xiangting Bernice Lin, Tih-Shih Lee, Yin Bun Cheung, Joanna Ling, Shi Hui Poon, Leslie Lim, Hai Hong Zhang, Zheng Yang Chin, Chuan Chu Wang, Ranga Krishnan, Cuntai Guan

**Affiliations:** 1 Neuroscience and Behavioral Disorders Program Duke University - National University of Singapore Medical School Singapore Singapore; 2 Singapore General Hospital Singapore Singapore; 3 Centre for Quantitative Medicine Duke University - National University of Singapore Medical School Singapore Singapore; 4 Center for Child Health Research University of Tampere and Tampere University Hospital Finland Finland Finland; 5 Singapore Clinical Research Institute Singapore Singapore; 6 Institute for Infocomm Research Agency for Science, Technology, and Research Singapore Singapore; 7 Rush Medical College of Rush University Chicago, IL United States; 8 Nanyang Technological University Singapore Singapore

**Keywords:** social anxiety, public speaking anxiety, exposure therapy, arousal feedback, randomized controlled trial

## Abstract

**Background:**

Exposure therapy is highly effective for social anxiety disorder. However, there is room for improvement.

**Objective:**

This is a first attempt to examine the feasibility of an arousal feedback–based exposure therapy to alleviate social anxiety symptoms in an analogue adult sample.

**Methods:**

A randomized, pilot, proof-of-concept trial was conducted to evaluate the acceptability, safety, and preliminary efficacy of our treatment program. Sessions were administered once a week for 4 weeks (1 hour each) to an analogue sample of 50 young adults who reported at least minimal social anxiety symptoms. Participants in both intervention and waitlist control groups completed assessments for social anxiety symptoms at the baseline, week 5, and week 10.

**Results:**

Most participants found the intervention acceptable (82.0%, 95% CI 69.0%-91.0%). Seven (14.9%, 95% CI 7.0%-28.0%) participants reported at least one mild adverse event over the course of study. No moderate or serious adverse events were reported. Participants in the intervention group demonstrated greater improvements on all outcome measures of public speaking anxiety from baseline to week 5 as compared to the waitlist control group (Cohen *d*=0.61-1.39). Effect size of the difference in mean change on the overall Liebowitz Social Anxiety Scale was small (Cohen *d*=0.13).

**Conclusions:**

Our results indicated that it is worthwhile to proceed to a larger trial for our treatment program. This new medium of administration for exposure therapy may be feasible for treating a subset of social anxiety symptoms. Additional studies are warranted to explore its therapeutic mechanisms.

**Trial Registration:**

ClinicalTrials.gov NCT02493010; https://clinicaltrials.gov/ct2/show/NCT02493010

## Introduction

### Background

Social anxiety disorder (SAD) is one of the most prevalent anxiety disorders [1], with the highest prevalence in high-income countries and an early age of onset globally [2]. It is chronic and associated with diminished quality of life [3-4]. SAD remains one of the most undiagnosed and undertreated mental disorders [5]. European data estimated that SAD cost €7277 million in direct health care costs in 2010, while indirect costs, factoring in absenteeism from work and early pension, amounted to €4806 million [6]. People affected by SAD reported impaired occupational productivity due to emotional problems and increased absences [7-8], and studies have found that they achieve lower educational attainment and earn wages 10% lower than a nonclinical population [9-10]. The core feature of SAD is a marked fear or anxiety about social interactions and performance situations in which one is exposed to possible evaluation by others, as described in the Diagnostic and Statistical Manual of Mental Disorders - 5th edition [11]. Generalized and nongeneralized subtypes of SAD can differentiated as follows: The generalized subtype is characterized by anxiety in most social situations, whereas the nongeneralized subtype is indicated by anxiety under specific circumstances such as public speaking [12-13]. Public speaking anxiety is found to be the most common characteristic of SAD, regardless of differences between the subtypes [14]. Accordingly, there is a need to target SAD with particular emphasis on public speaking anxiety.

Exposure therapy is the mainstay of SAD treatment. Individuals afflicted by SAD often do not seek treatment unless it is persistent, pervasive, or accompanied by other more acute psychiatric conditions [15-17]. Many avoid or are averse to seek help; therefore, self-administered technology offers a promising mode of delivery to raise treatment acceptance and accessibility. A meta-analysis of technology-assisted interventions for SAD suggested that internet-delivered cognitive behavioral therapy (iCBT) and virtual reality exposure therapy (VRET) are effective in relieving SAD symptoms [18]. Randomized controlled trials found VRET to be as efficacious as exposure group therapy [19-20] or traditional cognitive behavioral therapy (CBT) involving *in vivo* exposure [21-24]. Nonetheless, these interventions served only as alternative therapist-mediated treatment modalities in clinics. There are only a few rigorous studies on technology-assisted exposure therapy for SAD; as such, the reported positive outcomes remain preliminary at best. Mobile technology-based exposure therapy, which enables interventions to be taken home by those who need treatment support or find treatment in clinics difficult to tolerate, needs to be developed and tested.

Prevailing technology-based exposure therapies for SAD including VRETs are therapist-mediated and thus require manual adjustment of exposure parameters to suit individual needs. Anderson et al reported that therapists modified virtual audience reactions and environments physically depending on patients’ hierarchy of fears [20]. Kampmann et al reported that therapists using VRET manipulated the duration and difficulty level of interactions between patients and virtual humans, such as the degree of friendliness, gestures, or gender of avatars [22]. Since VRET served as adjunctive treatment under the therapist’s control in these studies, it is unclear whether an unmediated technology-only program is efficacious in reducing SAD symptoms. To our best knowledge, only two studies explored such an intervention. Kim et al conducted a 2-week, unmediated, mobile-based virtual reality program for patients with SAD and found the outcomes to be marginally significantly more positive among patients than those among normal age-matched controls [25]. Lindner et al evaluated a self-led one-session virtual reality program and found benefits for individuals with public speaking anxiety [26]. Instead of having therapists select exposure exercises for the individual, Lindner et al provided individuals with instructions within the program to self-direct and sort their own exposure exercises. Using a randomized controlled procedure, this study sought to clarify whether exposure therapy delivered by technology in the absence of therapist intervention could improve social anxiety symptoms.

Recent advances in the conceptualization of exposure therapy posit inhibitory learning as a more parsimonious theory to explain treatment effects and failure and advocate inhibitory learning techniques to optimize treatment effects among patients [27-28]. In cases of successful inhibitory learning-based exposure, fearful associations continue to exist but compete poorly with newly acquired associations. Thus, in theory, exposure therapies must aim to strengthen newly learned inhibitory associations for these associations to compete effectively with one’s previously held fearful associations. One means of strengthening newly learned inhibitory associations is to subject patients to prolonged and intense distress during exposure. Accordingly, we aimed to incorporate inhibitory learning into our intervention by means of sustaining distress to enhance exposure treatment outcomes.

Anxiety disorders have also been treated using biofeedback-based interventions. By convention, exposure therapies and biofeedback-based interventions progress along distinct lines of research: The former facilitates desensitization to a prespecified hierarchy of anxiety-provoking situations (eg, public speaking or eating in public) by *in vivo* or *in virtuo* exposure, and the latter targets anxiety in a predominantly broad manner by entraining anxiety regulation using physiological processes. Among psychiatric disorders treated by biofeedback-based interventions, anxiety disorders constituted the most commonly treated conditions, and electroencephalographic (EEG) biofeedback was the modality that received most attention [29]. Heart rate variability biofeedback-based programs were found to be associated with anxiety and stress reduction in a recent meta-analysis [30]. In general, biofeedback-based treatments involve a noninvasive procedure to train the patient to gain control over normally involuntary body functions. A patient’s physiological outputs (eg, brainwaves and heart rate) are detected, monitored, and processed electronically and then returned as feedback in different forms (eg, visual and auditory) to the same individual. Positive outcomes in the patient’s targeted physiology (eg, reduced physiological arousal) are yielded through constant positive feedback [31]. In other words, one’s anxiety is gradually reduced by receiving rewarding feedback every time he/she successfully lowers his/her anxiety during the course of the intervention.

Our study aimed to integrate biofeedback with portable hardware to enhance current technology-assisted exposure interventions for SAD. We argue that biofeedback technologies automate real-time modifications in exposure therapy as well as provide a means to sustain participant distress during exposure in accordance with inhibitory learning theory. Mechanistically different from typical biofeedback programs for anxiety, we designed and utilized a first-of-a-kind arousal feedback–based system that runs on inputs from individuals’ EEG and heart rate detection. An underlying arousal feedback–based machine learning algorithm combined EEG band powers, heart rate, and heart rate variability to influence difficulty parameters of the virtual exposure environment in real time. These dynamic changes facilitated retainment of discrepancy between participants’ actual and anticipated consequences, thereby sustaining inhibitory learning throughout the exposure session. Our combination of technologies forms an expedient means to deliver exposure therapy. Participants can be repeatedly exposed to newly learned safety associations and subjected to prolonged distress in a controlled virtual environment. Biofeedback technology helped calibrate and address individual differences in baseline anxiety and distress as well as automatize the exposure function through arousal feedback.

This was the first study to examine the viability of using biofeedback and portable technologies in delivering, personalizing, and optimizing exposure therapy in a laboratory setting. In particular, the purpose of our pilot trial was to investigate the feasibility of an arousal feedback–based exposure therapy to alleviate social anxiety symptoms, with particular emphasis on public speaking anxiety. We employed a randomized, waitlist-controlled design to evaluate the acceptability, safety, and preliminary efficacy of this treatment program. The study was conducted over a 4-week period with a 5-week follow-up of an analogue sample of 50 young adults who had public speaking anxiety complaints and reported at least minimal social anxiety symptoms.

### Specific Hypotheses

In this paper, we examined the feasibility of our exposure therapy with real-time arousal detection and feedback in reducing social anxiety symptoms. Specifically, for our primary objectives, we hypothesized that the acceptability rate of our program would be high, that adverse event rate would be low, and that participants in the intervention group would show greater improvement in social anxiety symptoms between baseline and week 5 assessments than the waitlist-control group. For our secondary objectives, we hypothesized that compared to the waitlist-control group participants, the intervention group participants would demonstrate greater improvements in public speaking anxiety, fear of negative evaluation, and self-statements made during public speaking from baseline to week 5.

## Methods

### Study Design

This was a randomized, waitlist-controlled, single-center open-label study. Participants completed assessments at baseline, week 5, and week 10. The intervention group attended intervention sessions from weeks 1 to 4, and the waitlist control group attended sessions between weeks 6 and 9. This study was approved by the Institutional Review Board of the National University of Singapore (reference code: B-14-098).

### Participants and Setting

The study was conducted from May 2016 to May 2017 at Duke-NUS Medical School, Singapore. Participants were recruited on a voluntary basis through various modes of advertising including clinician referrals, posters, newspapers, social media, institutional email notices (ie, Duke-NUS Medical School, National University of Singapore), and word of mouth. Interested participants were scheduled for a written informed consent and brief screening session. Each participant was told to complete an intervention schedule once a week over a 4-week period. Participants were also instructed to complete assessments at the baseline, week 5, and week 10. Reimbursement for time and transport was provided on a prorated basis upon completion or termination of the study. All participants were told that they may or may not benefit from participating in the intervention. Data collected were anonymized, and improvements reported were not associated with any personal benefit.

Eligibility was determined after consent was obtained. The inclusion criteria were age between 21 and 35 years, literacy in English and computer skills, absence of current or previous history of neuropsychiatric disorders, and willingness to be video recorded. Eligible participants had scores of ≥31 on the Liebowitz Social Anxiety Scale (LSAS) [32-33], ≥60 on the Public Speaking Anxiety Scale (PSAS) [34], and ≤8 on the Alcohol Use Disorders Identification Test [35]. Those who had suicidal ideation (indicated by item 9 of the Beck Depression Inventory - 2nd edition) [36], irregular heart rhythm, gross visual or hearing impairments, psychoactive medication, or concurrent psychotherapy were excluded from the study. Participants were also excluded if they were involved in any other longitudinal research study.

### Arousal Feedback–Based Exposure Therapy

**Figure 1:**
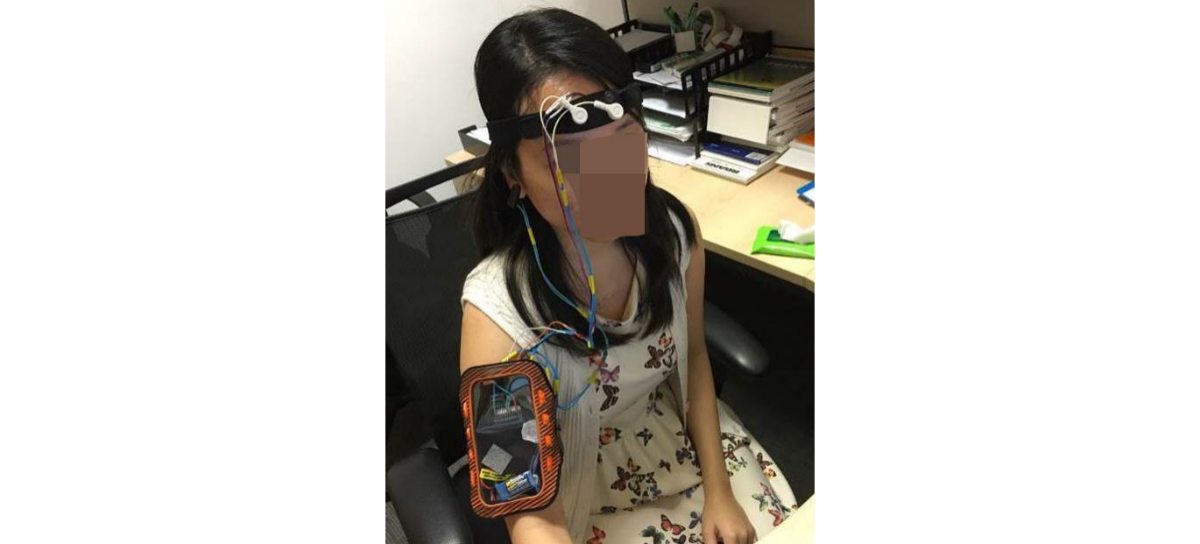
Locally developed, noninvasive headband.

The intervention was implemented using a locally developed, noninvasive portable headband (Figure 1) that connected wirelessly to a 14-inch commercial laptop via Bluetooth technology. The headband contained two EEG electrodes at the FP1 and FP2 locations, a heart pulse rate monitor, and an ear-clip with a grounding electrode. During training, the participant’s EEG waves and pulse rates were detected from the headband and transmitted to our system.

All participants completed the calibration process followed by the intervention process. During the calibration process, our system constructed a personalized arousal profile for each individual, based on unique physiological signatures (ie, EEG and photoplethysmogram patterns detected by our hardware) occurring during alternating induced periods of high and low arousal. The personalized arousal profile consisted of an adapted threshold, which was required to manipulate each individual’s exposure environment.

Our intervention consisted of 4 weekly sessions, each lasting about 60 minutes. Each session was conducted between 8.30 am and 8.30 pm on weekdays. During the intervention session, participants underwent three types of tasks: an interactive psychoeducation on screen, brief arousal control games, and arousal feedback–based speech tasks to a virtual audience. All materials were presented on the monitor of the same 14-inch laptop used to process physiological signals by the biofeedback system. Participants completed each session independently in a quiet room and were instructed to follow all instructions on the screen. The psychoeducation component delivered information about social anxiety and coping strategies and highlighted typical maladaptive thoughts and behaviors associated with particular anxiety-provoking social situations. To support the in-session learning material, participants were tested on key takeaways and tasked to identify their specific social anxiety-related concerns through pen-and-paper homework. Homework was to be completed every week before the next session. No formal assessment of homework performance was conducted.

**Figure 2:**
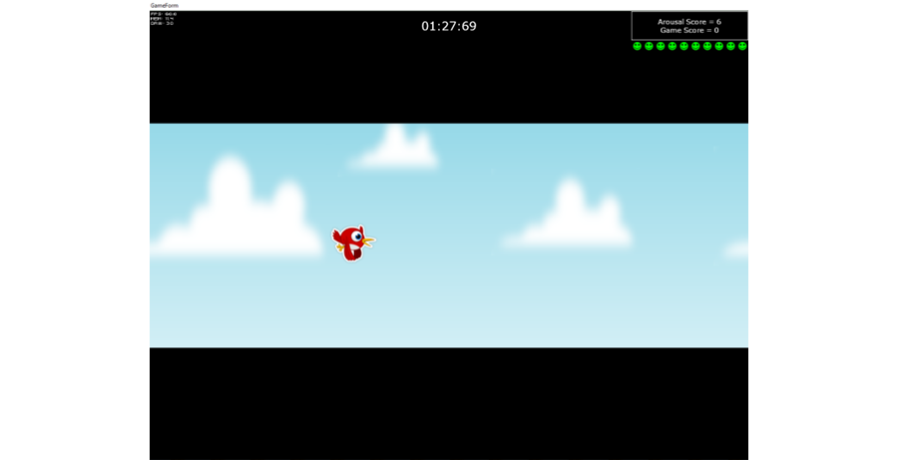
Brief arousal control game.

A brief arousal control game (Figure 2) was interspersed eight times between psychoeducation and six arousal feedback–based speech tasks. In this game, participants wore the headband and were instructed to increase and sustain the height of bird flight on the game interface by lowering their arousal levels. The bird avatar would fly lower when the arousal levels increased. This height of the bird flight served as nonthreatening, real-time feedback for participants to gain awareness of and actively manage their high arousal levels.

**Figure 3:**
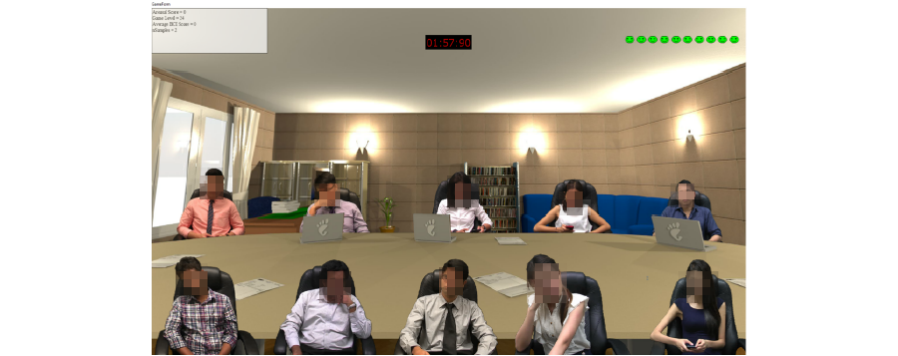
Virtual audience in arousal feedback–based speech task.

In the arousal feedback–based speech task, participants were tasked to deliver six 2-minute speeches to a virtual audience (Figure 3). The virtual audience was put together using prerecordings of real-life individuals who would display different types of facial expressions and body language, which simulated positive (smiling), neutral (straight face), or negative emotional expressions (disinterested, bored, sleeping, and looking at the mobile phone). While participants delivered their speech, they received concurrent feedback on their arousal levels indicated by an “arousal score” on screen. The arousal score was computed by our system’s algorithm in real time based on participants’ EEG and photoplethysmogram inputs, which modified the behavior of the virtual audience. An increasing proportion of the virtual audience exhibited negative expressions or body language, when participants’ arousal levels exceeded stipulated thresholds. Conversely, the virtual audience showed positive, affirming expressions when the arousal levels fell below the thresholds. As a result, the degree of anxiety provocation of virtual speech tasks was adapted to suit individual needs. Speech tasks were also made more difficult over the course of the intervention by increasing the size of the virtual audience, displaying less encouraging initial facial expressions of the virtual audience, presenting increasingly formal attire of the virtual audience, and assigning more demanding speech topics in a controlled step-wise manner. Difficulty of speech topics was predetermined by the degree of spontaneity and deliberation required: Personal topics were deemed easiest, followed by informative, persuasive, and impromptu topics. Participants were given 3 minutes to prepare prior to giving personal, informal, and persuasive speeches. No additional time was provided before participants gave impromptu speeches. Participants did not have control over these other parameters, which maintained difficulty in the speech tasks to support sustaining of inhibitory learning throughout the course of exposure sessions.

### Assessments at Baseline, Week 5, and Week 10

The LSAS is a validated and widely used 24-item questionnaire that assesses fear or anxiety and avoidance on a variety of social interaction and performance situations [32-33]. On a 4-point scale, participants rated their fear or anxiety (0=none, 3=severe) and avoidance (0=never, 3=usually) of each social situation. The scale yields an overall score by summing the item scores; higher scores indicate greater anxiety. An LSAS score of ≤30 indicates that SAD is unlikely. Internal consistency of the current sample was high (α=0.95).

The PSAS is a recently published 17-item self-rated questionnaire that measures cognitive, behavioral, and physiological manifestations of public speaking anxiety [34]. Participants rated positive and negative statements about giving speeches on a 5-point Likert-type scale (1=not at all, 5=extremely). The scale yields an overall score (items 6, 7, 8, 16, 17 are reverse coded); higher scores indicate greater anxiety. Internal consistency of the current sample was high (α=0.86).

The Fear of Negative Evaluation - Brief questionnaire (FNE-B) is a validated 12-item scale that assesses one’s fear of being judged negatively by others [37]. Participants rated positive and negative statements on a 5-point Likert-type scale (1=not at all characteristic of me, 5=extremely characteristic of me). The scale yields an overall score (items 2, 4, 7, and 10 are reverse coded); higher scores reflect greater fear. Internal consistency of the current sample was high (α=0.88).

The Self-Statements made during Public Speaking (SSPS) scale measures positive and negative thoughts about oneself during public speaking situations [38]. Participants rated 10 statements on a 6-point scale (0=do not agree, 5=agree extremely). The scale yields an overall score (items 1, 3, 5, 6, and 9 are reverse coded); higher scores reflect greater negativity. Internal consistency of the current sample was high (α=0.78).

Acceptability was defined as a rating of 5, 6, or 7 on “How would you rate the quality of the training system?” This was measured as part of a study-specific satisfaction and immersion questionnaire (SIQ; internal consistency: α=0.85) that examined participants’ attitudes toward the arousal feedback–based intervention on a 7-point Likert-type scale (1=poor, 7=excellent) postintervention. The form included a final open-ended question to capture comments or suggestions. All participants completed the SIQ postintervention.

### Sample Size

A total sample size of 41 participants was required to yield a precision (width of 95% CI) of approximately 12% in the proportion of participants who provide positive feedback on acceptability, assuming the true proportion is approximately 80%. Assuming an attrition rate of approximately 20%, a total sample size of 50 subjects was required. We simultaneously evaluated the preliminary efficacy of the training system to determine whether a larger-scale trial is warranted, by using Simon’s randomized selection design [39,40]. A total sample size of 50 would guarantee an 80% probability of correctly selecting the intervention arm as superior to the waitlist if it was truly superior by an effect size of 0.3 SD. If a positive difference was observed for preliminary efficacy in LSAS regardless of statistical significance, the intervention would be concluded to be promising and worthy of further investigation in a larger trial as per the randomized selection design.

### Randomization and Blinding

Randomization was performed in a 1:1 allocation ratio, using blocks of 10 with permuted subblocks of sizes 4 and 6, via a password-protected Web-based program. Block size was determined by the study statistician and not made known to clinical investigators or site personnel until after study closure.

### Statistical Analyses

Acceptability analysis was based on all enrolled subjects. Safety analyses were conducted for treated participants who received at least one intervention session. Efficacy analyses were intention-to-treat (ITT) and involved all randomized participants, with per protocol (PP) analysis conducted as sensitivity analysis. Acceptability analyses were rated by pooling responses on the SIQ question “How would you rate the quality of the training system?” from both intervention and waitlist control groups after receiving treatment. Missing acceptability assessment was imputed as “not acceptable.” Further complete-case sensitivity analyses of primary and secondary efficacy endpoints analyzed all participants with baseline assessments, accounting for missing data using a mixed-effects model with random subject intercepts, adjusted for group, time, and group-time interaction and with restricted maximum likelihood estimation.

Data for participants that found the training system acceptable and for whom the training system was safe were presented as Wilson score CIs. Preliminary efficacy evaluation was conducted using Cohen *d* for difference in change of LSAS total score from baseline to week 5 between the intervention and waitlist control groups.

Supplementary analyses compared median change and adjusted mean change (Multimedia Appendix 1). Sustainability of effect was described for the within-participant differences between preintervention and postintervention scores and loosely classified as nonreversion to preintervention levels or nonworsening of postintervention scores compared to preintervention scores. Pooled pre-post outcome scores from both the intervention and waitlist control groups were reported. Statistical analyses were performed using SAS software (v9.4; SAS Institute Inc, Cary, NC). All statistical procedures, including randomization and data analyses, were managed by an independent third party (Singapore Clinical Research Institute Private Limited, Singapore).

## Results

### Demographic and Baseline Characteristics

A total of 72 participants were screened, of whom 22 were excluded. Fifty were recruited and randomized into the intervention group (n=25) or waitlist control group (n=25). The majority of participants were female (n=37, 74%) and Chinese (n=42, 84%), with a mean age of 25.6 years. Baseline characteristics were similar between the intervention and waitlist control groups of the ITT population (Table 1) and between the ITT and PP populations (results not shown).

**Figure 4:**
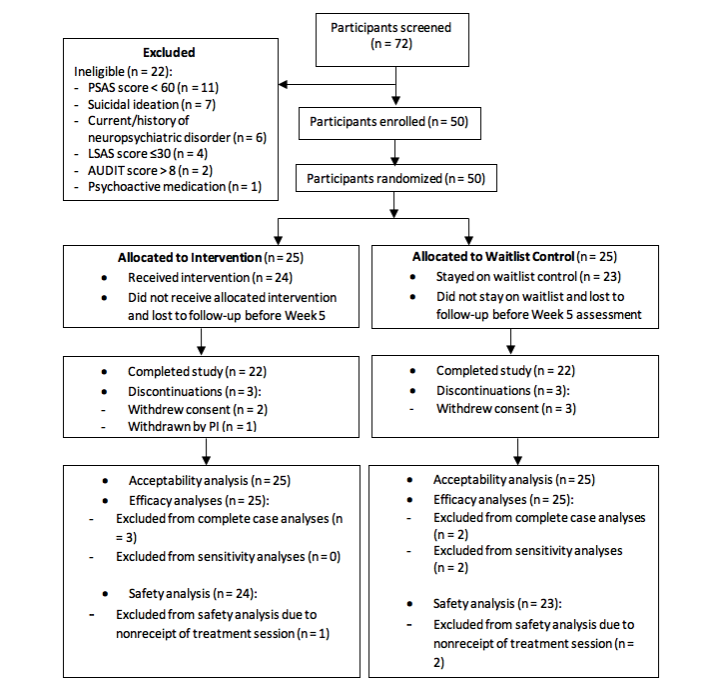
Consolidated Standards of Reporting Trials (CONSORT) diagram. AUDIT: Alcohol Use Disorders Identification Test; LSAS: Liebowitz Social Anxiety Scale; PSAS: Public Speaking Anxiety Scale.

A majority (n=44, 88%) of the participants received all four intervention sessions; in addition, 45 (90%) completed week 5 assessments and 44 (88%) completed week 10 assessments. There were five (10%) withdrawals initiated by participants due to their inability to commit to the study and one (2%) by the investigator due to an ear condition that interfered with hardware administration. Three withdrawals occurred before intervention, and three occurred at weeks 1, 2, and 3. The Consolidated Standards of Reporting Trials (CONSORT) flow diagram is shown in Figure 4. Six cases of incomplete assessments were considered protocol violations and excluded from PP analyses. The number of participants in ITT or PP analyses, at each time point, was reported in Multimedia Appendix 1.

**Table 1:** Demographic and baseline characteristics.

Characteristic		Intervention (n=25)	Waitlist control (n=25)	Total (N=50)
Age (years), mean (SD)		24.2 (3.23)	27.0 (4.19)	25.6 (3.96)
Female, n (%)		17 (68.0)	20 (80.0)	37 (74.0)
**Ethnicity, n (%)**				
	Chinese	20 (80)	22 (88)	42 (84)
	Other	5 (20)	3 (12)	8 (16)
**Current education, n (%)**				
	Secondary education	0 (0)	1 (4.0)	1 (2.0)
	Preuniversity	0 (0)	2 (8.0)	2 (4.0)
	Currently in/graduated from university	25 (100)	22 (88.0)	47 (94.0)
BDI-II^a^ total score, mean (SD)		12.0 (7.52)	10.0 (9.10)	11.0 (8.32)
AUDIT^b^ total score, mean (SD)		1.1 (1.59)	1.9 (2.22)	1.5 (1.95)
LSAS^c^ total score, mean (SD)		68.8 (20.63)	69.7 (21.61)	69.3 (20.91)
PSAS^d^ total score, mean (SD)		67.0 (4.53)	68.4 (5.92)	67.7 (5.27)
FNE-B^e^ total score, mean (SD)		45.6 (7.08)	43.6 (6.65)	44.6 (6.88)
SSPS^f^ total score, mean (SD)		26.5 (5.67)	24.1 (7.35)	25.4 (6.57)

^a^BDI-II: Beck Depression Inventory (2nd edition).

^b^AUDIT: Alcohol Use Disorders Identification Test.

^c^LSAS: Liebowitz Social Anxiety Scale.

^d^PSAS: Public Speaking Anxiety Scale.

^e^FNE-B: Fear of Negative Evaluation - Brief questionnaire.

^f^SSPS: Self-Statements made during Public Speaking scale.

### Acceptability

Most participants (82.0%, 95% CI 69.0%-91.0%) found the training system acceptable. The remaining, including 6 missing assessments, were classified as “not acceptable.”

### Safety

Seven (14.9%, 95% CI 7.0%-28.0%) participants reported at least one adverse event over the course of the study. There were a total of eight mild adverse events including eye strain (n=1), itch on forehead and scalp (n=1), headache (n=3), and dizziness (n=3). No moderate or serious adverse events were reported.

### Change in Efficacy Scores

Table 2 showed the mean total LSAS, PSAS, FNE-B, and SSPS scores at weeks 0 and 5 for the intervention and waitlist control groups. Mean total LSAS scores between weeks 0 and 5 decreased by 1.5 points in the intervention group and increased by 0.8 points in the waitlist control group. The Cohen *d* effect size for differences in mean change in total LSAS scores between groups was 0.13 points. Mean change scores of PSAS, FNE-B, and SSPS between weeks 0 and 5 ranged from 3.0 to 10.2 points in the intervention group and –0.9 to 1.3 points in the waitlist control group. The Cohen *d* estimates of differences in mean change scores of PSAS, FNE-B, and SSPS between groups were 1.39, 0.61, and 0.79, respectively. Similar results were found in the PP population (results not shown).

**Table 2:** Mean total efficacy scores (SD), mean change scores (SD), and effect sizes of differences in mean changes between weeks 0 and 5. Change in total score = week 0 total score – week 5 total score. A positive change in total score indicates improvement (a reduction in symptoms/scores).

Measures	Group						Cohen *d* effect size, mean (95% CI)		
	Intervention			Waitlist							
	Week 0	Week 5	Change	Week 0	Week 5	Change			
LSAS^a^	68.8 (20.63)	67.7 (20.87)	1.5 (20.54)	69.7 (21.61)	72.2 (21.32)	–0.8 (14.55)			0.13 (–0.47 to 0.72)
PSAS^b^	67.0 (4.53)	56.9 (9.83)	10.2 (8.56)	68.4 (5.92)	67.7 (7.49)	1.3 (3.07)			1.39 (0.72 to 2.05)
FNE-B^c^	45.6 (7.08)	43.0 (9.20)	3.0 (8.10)	43.6 (6.65)	44.5 (7.51)	–0.9 (4.38)			0.61 (0.00 to 1.22)
SSPS^d^	26.5 (5.67)	21.2 (8.24)	6.2 (8.42)	24.1 (7.35)	24.1 (8.61)	–0.0 (7.46)			0.79 (0.17 to 1.41)

^a^LSAS: Liebowitz Social Anxiety Scale.

^b^PSAS: Public Speaking Anxiety Scale.

^c^FNE-B: Fear of Negative Evaluation - Brief questionnaire.

^d^SSPS: Self-Statements made during Public Speaking scale.

### Supplementary and Sensitivity Analyses

Unadjusted and adjusted results for efficacy measures were qualitatively the same as results for Cohen *d*; results from sensitivity analyses were similar to the unadjusted results (Multimedia Appendix 1).

### Sustainability Analysis

The waitlist control group received the intervention from weeks 6 to 9, with mean changes in efficacy measures between weeks 5 and 10 ranging from 2.2 to 6.5. Corresponding mean changes in efficacy measures between weeks 5 and 10 of the intervention group ranged from 0.9 to 10.0. Pooled pre- and postintervention changes of both arms showed improvement (changes in mean point estimates of 2.6-8.2) on all efficacy outcomes (Table 3). Exploratory analyses found secondary efficacy outcomes of PSAS, FNE-B, and SSPS to be significant (*P*<.05).

**Table 3:** Pooled pre- and postintervention efficacy scores. The pooled pre- and post- intervention change score is calculated as the sum of change scores of both arms, where the intervention arm change score is scores of week 0 – week 5, and waitlist control arm change score is score of week 5 – week 10; a positive change indicates improvement (a reduction in symptoms).

Measures		Mean change (SD)	*P* value^a^
**Primary**			
	LSAS^b^	4.0 (21.66)	.23
**Secondary**			
	PSAS^c^	8.2 (7.50)	<.001
	FNE-B^d^	2.6 (6.59)	.01
	SSPS^e^	5.5 (7.80)	<.001

^a^*P* value from one-sample *t* test.

^b^LSAS: Liebowitz Social Anxiety Scale.

^c^PSAS: Public Speaking Anxiety Scale.

^d^FNE-B: Fear of Negative Evaluation - Brief questionnaire.

^e^SSPS: Self-Statements made during Public Speaking scale.

## Discussion

The study results indicated that the arousal feedback–based exposure therapy was acceptable and safe. Improvements in the PSAS, FNE-B, and SSPS scores, which were sustained over a follow-up 5-week period, suggested that our intervention might be efficacious in alleviating adult public speaking anxiety. Our findings did not provide strong support for the efficacy of our intervention in reducing overall social anxiety symptoms on the LSAS. Caution is also needed when interpreting the difference in change scores, as the randomized selection design was only meant to identify intervention that is worthy of further research instead of providing confirmation of efficacy. Overall, our preliminary findings indicated that it is worthwhile to proceed with a larger trial.

The intervention was safe and acceptable. Majority of the few complaints concerned prolonged use of hardware rather than treatment material and software. Young adults today are mobile- and technologically savvy but not yet accustomed to biofeedback technologies. Thus, discomfort with system usage was not unforeseeable. Regarding the treatment material, study participants indicated that “simulations did not feel real” and “having real life audience would be helpful” in the feedback comments. Although the levels of immersive exposure experience may not reach those elicited by *in vivo* speech tasks [41], there were no significant differences found on self-reported anxiety, heart rate, heart rate variability, and saliva cortisol levels when comparing *in vivo* and *in virtuo* exposures [42]. Performance-based social anxiety including public speaking anxiety is also significantly associated with physiological hyperarousal [43]. Thus, our findings demonstrated the relevance of targeting the physiological level in exposure therapies for SAD. The arousal feedback–based exposure therapy that targeted physiological processes during public speaking in a laboratory setting could alleviate public speaking anxiety despite subjective negative perceptions of immersion. In addition, our computer display was sufficient to elicit ameliorating effects on public speaking anxiety symptoms; it is possible that by using hardware of higher resolution and comfort, specifically a virtual reality headset, the degree of immersive experience and thus benefits of exposure could be increased.

Our study recruited an analogue adult sample and did not include formal clinical diagnoses. Nonetheless, participants enrolled in this study were not unlikely to have SAD, as indicated by baseline scores of ≥31 on the LSAS [32]. Majority of participants (64%) who completed all parts of the study had moderate to very severe SAD symptoms, reporting scores between 60 and 118 points on the LSAS at baseline. Although improvements in social anxiety were observed at postintervention, the effect size of change on the LSAS was small (ie, Cohen *d*=0.13), and the corresponding absolute outcome scores remained at subclinical levels. It was possible that this negative result was due to the relatively small sample size and thus poor statistical power.

Another more probable explanation for the negative result found on the LSAS was that our intervention helped specifically in addressing public speaking anxiety, which constitutes a subset of SAD symptoms. Effect sizes of improvements on secondary outcome measures, ie, the PSAS, FNE-B, and SSPS scale, ranged from moderate to large (ie, Cohen *d* ranged from 0.61 to 1.39). The differential findings between overall social anxiety and secondary measures of public speaking anxiety provided preliminary support for the efficacy of our arousal feedback–based exposure therapy in reducing specific public speaking anxiety symptoms. However, our intervention had multiple components (eg, inclusion of psychoeducation, which have known effects); therefore, dismantling studies are necessary to explore the potential mechanisms for treatment efficacy.

Our findings further concurred with extant literature indicating that SAD should be differentiated and treated according to a more severe generalized subtype or a less severe nongeneralized subtype encompassing public speaking anxiety [10,44]. Our intervention demonstrated greater potential efficacy in ameliorating specific symptoms of public speaking anxiety than in the overall syndrome of social anxiety. Interestingly, some researchers had suggested that public speaking anxiety could be a distinct SAD subtype of its own [12]. Others proposed defining SAD as a continuum of clinical severity based on the number of feared social situations [45]. Although the characterization of public speaking anxiety in SAD remains contentious, public speaking continues to be a major source of anxiety in SAD and warrants intervention.

Our exposure therapy integrated a key feature of inhibitory learning (ie, distress tolerance) to enhance treatment effects. However, we did not maximize violations of participants’ fear-based expectancies for harm or provide multiple contexts to facilitate the decontextualization of newly learned inhibitory associations [46]. In theory, participants should be constantly reminded of the discrepancy between actual and anticipated consequences. They should be continually exposed to new and actual safety associations (eg, no repercussions from stumbling in speech) as opposed to anticipated feared associations (eg, “people make fun of me when I stumble in speech”). Diverse contexts are also necessary to increase one’s mental accessibility beyond the treatment session to new associations learned. By expanding the range of contextual cues that are associated with new learning, freshly acquired inhibitory associations can be strengthened in-session. The exclusion from intervention of other anxiety-provoking social situations could also partially explain why benefits were found in public speaking anxiety-specific measures but not on the overall social anxiety measure. To better align our arousal feedback–based exposure therapy with inhibitory learning, violations of participants’ fear-based expectancies for harm need to be incorporated and maximized. Our intervention can be extended to treat other domains of SAD by developing and targeting other anxiety-provoking social situations.

Set against traditional habituation-based exposure therapies, exposure treatments based on the inhibitory learning model do not necessitate fear reduction during exposure to produce posttreatment fear extinction. Although habituation models suggest that fearful associations (eg, “people hate to hear me speak”) must be eliminated altogether for treatment efficacy, inhibitory learning models postulate that successful exposure occurs even when fearful associations are not eliminated. Although described initially as different theories of exposure therapy [27], the distinction between habituation and inhibitory learning models remains arbitrary. Benito and Walther clarified that habituation does not necessarily entail replacement of feared associations with newly learned safety associations, contrary to what was conceived in its parent Emotional Processing Theory [47,48]. The authors argued that habituation is a “therapeutic process...somewhat agnostic to the precise underlying mechanism.” Fear activation and minimization of maladaptive anxiety-reducing behaviors are imperative to optimize exposure therapy. These habituation-based elements are analogous to sustaining distress and inducing tolerance during inhibitory learning-based exposure. One postulated difference between the two models appears to lie in the disparity between within-session and between-session anxiety reduction. Specifically, inhibitory learning happens when between-session anxiety reduction occurs in the absence of within-session anxiety reduction, whereas both within- and between-session anxiety reductions ought to take place to elicit habituation-based treatment effects. Unfortunately, within- and between-session anxiety reductions have been traditionally difficult to study. For instance, it is challenging to operationalize within-session anxiety reduction as well as to examine between-session anxiety reduction, given confounding factors such as increasing exposure task difficulty over the course of intervention [47]. Bearing this difficulty in mind, laboratory-based experimental studies need to be carefully designed to investigate therapeutic mechanisms of inhibitory learning techniques in the context of our exposure therapy as well as to differentiate between habituation-based and inhibitory learning-based exposure therapeutic mechanisms, in general.

Some limitations restricted the generalizability of our study findings to patients with SAD, including the recruitment of an analogue subclinical adult sample and a lack of objective outcome measures (eg, measuring performance and arousal during speech to a real audience). This study employed self-rated measurement tools that could be confounded by participant bias or motivation to alleviate social anxiety. However, this was unlikely, given the differential outcomes of the overall social anxiety vis-à-vis specific public speaking anxiety measures. Nonetheless, a replication study investigating the effects of arousal feedback–based exposure therapy using a clinically representative sample and objective assessment tools should be conducted. Future research should also consider conducting active-control studies to tease out the differential effects between arousal feedback–based exposure therapy and therapist-mediated exposure therapy.

In conclusion, it is worthwhile to proceed to a larger trial. This pilot proof-of-concept study is a first attempt to establish the acceptability, safety, and potential efficacy of an arousal feedback–based exposure therapy for an analogue adult sample in order to reduce a subset of social anxiety symptoms. Our findings contribute to a growing body of literature on incorporating technology into mental health care services to improve treatment accessibility. Technology-assisted exposure therapies were previously found to be more cost-effective and amenable to therapists or clients than CBT for SAD [21,24]. Importantly, although we do not purport the displacement of pharmacotherapy and CBT as first-line treatments, our unmediated arousal feedback–based exposure therapy circumvents limitations in personalization of existing VRETs. Thus, it serves as an enhanced complement to current treatment modalities for SAD.

## Appendix

Multimedia Appendix 1 Statistical analysis.

Multimedia Appendix 2 CONSORT‐EHEALTH checklist (V 1.6.1).

## Abbreviations

AUDIT: Alcohol Use Disorders Identification Test
BDI-II: Beck Depression Inventory (2nd ed)
CBT: cognitive behavioral therapy
EEG: electroencephalogram
FNE-B: Fear of Negative Evaluation - Brief questionnaire
iCBT: internet-delivered cognitive behavioral therapy
ITT: intention to treat
LSAS: Liebowitz Social Anxiety Scale
PP: per protocol
PSAS: Public Speaking Anxiety Scale
SAD: social anxiety disorder
SIQ: satisfaction and immersion questionnaire
SSPS: Self-Statements made during Public Speaking
VRET: virtual reality exposure therapy
