# Supplementary material for: Exposure Therapy With Personalized Real-Time Arousal Detection and Feedback to Alleviate Social Anxiety Symptoms in an Analogue Adult Sample: Pilot Proof-of-Concept Randomized Controlled Trial
Source: JMIR Ment Health. 2019 Jun 14;6(6):e13869. doi: 10.2196/13869 (PMC6594210; doi:10.2196/13869)
Supplement: Multimedia Appendix 1 [file mental_v6i6e13869_app1.docx]

**Appendix A**

Statistical analysis:

Supplementary analyses compared median change using Hodges-Lehmann estimator (median of pairwise differences) with asymptotic Moses CIs and Mann-Whitney U test, mean change using ANOVA and ANCOVA (adjusted for group, baseline score, and group-by-baseline score interaction), Table A2.

Table A1. Number of participants analyzed at each time point (complete efficacy assessments)

|  |  |  | **Intention-to-treat** | | **Per protocol** | | **Sensitivity** | |
| --- | --- | --- | --- | --- | --- | --- | --- | --- |
| **Endpoint** | **Time** | **Assessment** | **Intervention** | **Waitlist** | **Intervention** | **Waitlist** | **Intervention** | **Waitlist** |
| **Primary** | | | | | | | | |
| **LSAS** | **Week 0** | 1 | 25 | 25 | 22 | 22 | 25 | 25 |
|  | **Week 5** | 2 | 22 | 23 | 22 | 22 | 25 | 25 |
|  | **Week 10** | 3 | 22 | 22 | 22 | 22 | 25 | 25 |
| **Secondary** | | | | | | | | |
| **PSAS** | **Week 0** | 1 | 25 | 25 | 22 | 22 | 25 | 25 |
|  | **Week 5** | 2 | 22 | 23 | 22 | 22 | 25 | 25 |
|  | **Week 10** | 3 | 22 | 22 | 22 | 22 | 25 | 25 |
| **FNE-B** | **Week 0** | 1 | 25 | 23 | 22 | 22 | 25 | 23 |
|  | **Week 5** | 2 | 22 | 23 | 22 | 22 | 25 | 23 |
|  | **Week 10** | 3 | 22 | 22 | 22 | 22 | 25 | 23 |
| **SSPS** | **Week 0** | 1 | 25 | 23 | 22 | 22 | 25 | 23 |
|  | **Week 5** | 2 | 22 | 23 | 22 | 22 | 25 | 23 |
|  | **Week 10** | 3 | 22 | 22 | 22 | 22 | 25 | 23 |

Table A2. Supplementary and sensitivity efficacy results of change^c^ in total score

| **Endpoint** | **Median^d^ change** | ***p*-value^e^** | **Unadjusted^f^**  **mean change** | **Adjusted^g^**  **mean change** | **Sensitivity^h^**  **mean change** |
| --- | --- | --- | --- | --- | --- |
| **Primary** |  |  |  |  |  |
| **LSAS** | 3.5 (-9.0, 13.0) | .532 | 2.2 (-8.43, 12.90) | 3.0 (-6.83, 12.90) | 2.7 (-8.95, 14.36) |
| **Secondary** |  |  |  |  |  |
| **PSAS** | 9.0 (4.0, 12.0) | <.001 | 8.8 (5.00, 12.66) | 8.6 (4.65, 12.59) | 9.1 (4.98, 13.14) |
| **FNE-B** | 3.0 (0.0, 6.0) | .092 | 4.0 (0.07, 7.85) | 3.5 (-0.45, 7.44) | 3.8 (-0.45, 8.05) |
| **SSPS** | 6.0 (2.0, 11.0) | .002 | 6.3 (1.50, 11.05) | 4.7 (-0.16, 9.63) | 5.7 (0.75, 10.73) |

^c­­^ change = Week 0 total score – Week 5 total score, a positive change indicates improvement (a reduction in symptoms); ^d^ Hodges-Lehmann estimator (median of pairwise differences) with asymptotic Moses confidence intervals; ^e^ Mann-Whitney U test; ^f^ ANOVA with change as dependent variable and group as independent variable; ^g^ ANCOVA with change as dependent variable and group, baseline, and group-by-baseline interaction as independent variables ^h^ Sensitivity analysis of missing values for all participants in population using mixed model with raw score as dependent variable and group, time, group-by-time interaction as fixed effects and random intercept
